# Correction to “Determinants of Healthcare Costs in Individuals With Down Syndrome: A Systematic Review”

**DOI:** 10.1002/hsr2.73116

**Published:** 2026-08-26

Shiva Tolouei Rakhshan, Sarah Byford, Mahya Razimoghadam, Fardin Moradi, Shahin Soltani. *Determinants of Healthcare Costs in Individuals With Down Syndrome: A Systematic Review*. **Health Science Reports**. 2026;9, https://doi.org/10.1002/hsr2.72297.

The affiliations of the authors were incorrectly presented in the published article. The correct author affiliations should be as follows:

Shiva Tolouei Rakhshan^1,^ ^2^ | Sarah Byford^3^ | Mahya Razimoghadam^4,^ ^5^ | Fardin Moradi^6^ | Shahin Soltani^1,^ ^7^

^1^ Research Center for Environmental Determinants of Health (RCEDH), Health Institute, Kermanshah University of Medical Sciences, Kermanshah, Iran

^2^ Scientific Association of Hospital Administration, Tehran University of Medical Sciences (TUMS), Tehran, Iran

^3^ King's College London, London, UK

^4^ National Center for Health Insurance Research, Tehran, Iran

^5^ Department of Health Management, Policy and Economics, School of Public Health, Tehran University of Medical Sciences, Tehran, Iran

^6^ Medical Education Department Center (EDC), Kurdistan University of Medical Sciences, Sanandaj, Iran

^7^ Students Research Committee, Kermanshah University of Medical Sciences, Kermanshah, Iran

The author names and affiliations should therefore appear in the published article according to the corrected numbering above. The correction concerns the presentation and numbering of the authors' affiliations only; no other aspect of the article is affected.

We apologize for this error.

